# Protein disulfide-isomerase A4 confers glioblastoma angiogenesis promotion capacity and resistance to anti-angiogenic therapy

**DOI:** 10.1186/s13046-023-02640-1

**Published:** 2023-03-30

**Authors:** Zewei Tu, Chong Wang, Qing Hu, Chuming Tao, Zhansheng Fang, Li Lin, Kunjian Lei, Min Luo, Yilei Sheng, Xiaoyan Long, Jingying Li, Lei Wu, Kai Huang, Xingen Zhu

**Affiliations:** 1 Department of Neurosurgery, The Second Affiliated Hospital of Nanchang University, Jiangxi 330006 Nanchang, P. R. China; 2 Jiangxi Key Laboratory of Neurological Tumors and Cerebrovascular Diseases, Jiangxi 330006 Nanchang, P. R. China; 3 Institute of Neuroscience, Nanchang University, Jiangxi 330006 Nanchang, P. R. China; 4 JXHC Key Laboratory of Neurological Medicine, Jiangxi 330006 Nanchang, P. R. China; 5 The Huan Kui Medical College of Nanchang University, Jiangxi 330006 Nanchang, P. R. China; 6 East China Institute of Digital Medical Engineering, Shangrao, China; 7 Department of Comprehensive Intensive Care Unit, The Second Affiliated Hospital of Nanchang University, Nanchang, P. R. China

**Keywords:** Protein disulfide-isomerase A4 (PDIA4), Glioblastoma (GBM), Angiogenesis, Endoplasmic reticulum stress (ERS), X-box binding protein 1 (XBP1)

## Abstract

**Introduction:**

Increasing evidence has revealed the key activity of protein disulfide isomerase A4 (PDIA4) in the endoplasmic reticulum stress (ERS) response. However, the role of PDIA4 in regulating glioblastoma (GBM)-specific pro-angiogenesis is still unknown.

**Methods:**

The expression and prognostic role of PDIA4 were analyzed using a bioinformatics approach and were validated in 32 clinical samples and follow-up data. RNA-sequencing was used to search for PDIA4-associated biological processes in GBM cells, and proteomic mass spectrum (MS) analysis was used to screen for potential PDIA4 substrates. Western blotting, real-time quantitative polymerase chain reaction (RT-qPCR), and enzyme-linked immunosorbent assays (ELISA) were used to measure the levels of the involved factors. Cell migration and tube formation assays determined the pro-angiogenesis activity of PDIA4 in vitro. An intracranial U87 xenograft GBM animal model was constructed to evaluate the pro-angiogenesis role of PDIA4 in vivo.

**Results:**

Aberrant overexpression of PDIA4 was associated with a poor prognosis in patients with GBM, although PDIA4 could also functionally regulate intrinsic GBM secretion of vascular endothelial growth factor-A (VEGF-A) through its active domains of Cys-X-X-Cys (CXXC) oxidoreductase. Functionally, PDIA4 exhibits pro-angiogenesis activity both in vitro and in vivo, and can be upregulated by ERS through transcriptional regulation of X-box binding protein 1 (XBP1). The XBP1/PDIA4/VEGFA axis partially supports the mechanism underlying GBM cell survival under ER stress. Further, GBM cells with higher expression of PDIA4 showed resistance to antiangiogenic therapy in vivo.

**Conclusions:**

Our findings revealed the pro-angiogenesis role of PDIA4 in GBM progression and its potential impact on GBM survival under a harsh microenvironment. Targeting PDIA4 might help to improve the efficacy of antiangiogenic therapy in patients with GBM.

**Supplementary Information:**

The online version contains supplementary material available at 10.1186/s13046-023-02640-1.

## Introduction

Glioblastoma (GBM) multiforme poses an insurmountable challenge due to its poor clinical outcome and median overall survival (OS) of approximately 7 months without treatment [1, 2]. GBM is biologically and pathologically characterized by aggressive infiltrating growth, immunological suppression by the microenvironment, and extensive neovascularization, all of which contribute to the evasion of GBM cells from immune clearance, increased survival in a harsh microenvironment, and poor prognosis and a high recurrence rate [3, 4]. General treatment of GBM includes surgical resection combined with postoperative chemotherapy and/or radiation therapy, which have been the main therapy strategies for several decades [2, 4]. Although, despite the advances in biomedical technology in recent years, novel treatment approaches and the discouraging prognosis of patients with GBM have not been significantly improved. Therefore, the search for new targets and the availability of new treatment strategies for GBM is critical and urgently needed for both patients and clinicians.

Protein disulfide isomerases (PDI) are a series of multifunctional proteins that maintain intracellular homeostasis by regulating the formation of disulfide bonds and correct protein folding, and have been reported to be involved in carcinogenesis and progression [5]. These enzymes have oxidoreductase and protein chaperone activities, and increasing evidence has indicated that PDI functions play a vital role in cancer proliferation, metastasis, drug resistance, and immune escape activities [6–10]. For example, PDIA3 expression of GBM cells can enhance the pro-tumor ability of macrophage/microglia [11], and knock-out Pdia3 in mice CDD8 + T cells can promote the GBM-killing capacity of T cells in vivo [10]; PDIA4 and PDIA6 contributes to the cisplatin resistance of lung adenocarcinoma [6]; PDIA6 also regulates the EGFR-dependent migratory and invasive abilities of GBM cells [8].

Protein disulfide isomerase A4 (PDIA4), also known as ERp72, is a member of the PDI family and consists of 645 amino acids, including three classical CXXC active domains [12]. PDIA4 is a component of the multiprotein chaperone complex, combining with BiP, PDI, ERp29, and Grp94, which regulates the redox state of proteins and cellular secretion [13]. Multiple studies have determined that PDIA4 is overexpressed in various cancer cell lines and clinical samples, and is highly associated with the clinical prognosis of cancer patients, which suggests that PDIA4 could be a vital biomarker and target for cancer treatment [14, 15]. Furthermore, the knockdown of PDIA4 expression in cancer cells interferes with cell growth, cell apoptosis, and increased chemotherapy sensitivity [6, 9, 16–18]. However, the detailed role and mechanisms of PDIA4 activity in GBM have not been elucidated, and its functions in GBM require further investigation.

In the present study, we demonstrated that PDIA4 is involved in GBM angiogenesis by regulating GBM-derived VEGFA secretion via its CXXC active domains. Furthermore, as an endoplasmic reticulum (ER) stress regulator, PDIA4 is up-regulated under ER stress conditions through XBP-1 transcriptional activity to enhance GBM-derived VEGFA secretion. The XBP1/PDIA4/VEGFA regulatory axis may be responsible for how GBM cells obtain nutrients within the poor tumor microenvironment by accelerating angiogenesis. Additionally, this study also confirmed that in xenograft GBM mice models, higher GBM expression of PDIA4 presented a significant antiangiogenic therapy resistant phenotype. Our findings not only reveal the mechanisms underlying PDIA4 involvement in GBM progression, but also propose targeting of PDIA4 as a promising precision therapy approach for patients with GBM.

## Methods and materials

### Public GBM data sources

To perform a pan-cancer analysis of the expression of PDIA4, we integrated the transcriptomic data of PDIA4 from 33 cancers and the corresponding normal tissues of The Cancer Genome Atlas (TCGA) and the Genotype-Tissue Expression (GTEx) data sets. Four independent public GBM cohorts (TCGA-GBM, *n* = 163; CGGA-seq1, *n* = 135; CGGA-seq2, *n* = 214; GSE16011, *n* = 147) were downloaded from The Cancer Genomic Atlas (TCGA, https://portal.gdc.cancer.gov/) [19], the Chinese Glioma Genomic Atlas (CGGA, http://www.cgga.org.cn/) [20], and the Gene Expression Omnibus (GEO, https://www.ncbi.nlm.nih.gov/gds) repository. Associated clinical information was also obtained from these databases and from a previous publication [21]. The single cell RNA sequencing data of four patients with GBM containing 3589 cells were also acquired from the GEO database (GSE84465) [22].

### GBM sample collection

A total of 32 human GBM samples with adjacent paired tissues were resected from inpatients who underwent treatment in the Neurosurgery Department of The Second Affiliated Hospital of Nanchang University (NCUSAH) from 2019 to 2022. The tumor samples were stored in liquid nitrogen after resection from patients with GBM immediately. Informed consent was obtained from the patients enrolled in this study. The use of clinical samples was approved by the NCUSAH Medical Ethics Committee. The collection and use of clinical samples was in strict accordance with the Helsinki guidelines.

### Cell lines and cell culture

The human GBM cell lines LN229, U118, U87, U251, and T98G were purchased from the American Type Culture Collection (ATCC, USA). All GBM cell lines were cultured with Dulbecco’s Modified Eagle’s Medium (DMEM, Gibco, USA) containing 10% fetal bovine serum (FBS, Gibco, USA) and antibiotics (100 units/mL penicillin and 100 mg/mL streptomycin, Gibco, USA) in a 37 °C incubator with 5% CO_2_ and 100% humidity. The human astrocyte cell line HA cells (ScienCell, Cat. No. #1800) was cultured in basal astrocyte medium (ScienCell, Cat. No. #1801) supplemented with 2% FBS and 1% astrocyte growth supplement (ScienCell, Cat. No. #1852). Human umbilical vein endothelial cells (HUVEC, Cat. No. #8000, ScienCell) were cultured with Endothelial Cell Medium (ECM, Cat. No. #1001, ScienCell) containing 1% endothelial cell growth supplement (ECGS, Cat. No. #1052, ScienCell), 5% FBS (Cat. No. #0025, ScienCell) and 1% antibiotic solution (P/S, Cat. No #0503, ScienCell) in a 37 °C incubator.

### Antibodies and agents

Antibodies and agents used in this study were as follows: anti-PDIA4 (Proteintech, Cat. No. #14,712–1-AP), anti-ATF6 (Proteintech, Cat. No. #24,169–1-AP), recombinant anti-XBP1 antibody (Abcam, Cat. No. #ab220783), anti-VEGFA (Proteintech, Cat. No. #19,003–1-AP), anti-Flag (Proteintech, Cat. No. 20543–1-AP), anti-HA (Proteintech, Cat. No. #66,006–2-Ig), anti-GAPDH (Proteintech, Cat. No. #10,494–1-AP), anti-HIF-1α (Proteintech, Cat. No. #20,960–1-AP), anti-β-Actin (Proteintech, Cat. No. #81,115–1-RR), anti-CD31 (Proteintech, Cat. No. 311265–1-AP), tunicamycin (Cat. No. #HY-A0098, MedChemExpress), temozolomide (Cat. No. HY-17364, MedChemExpress), human-VEGFA (Cat. No. HZ-1038, human VEGF-165 recombinant protein, Proteintech), and bevacizumab (Cat. No. #HY-P9906, MedChemExpress).

### Western blotting assay

The total protein of GBM cells was extracted using RIPA lysis buffer (Beyotime, Cat. No. P0013B, China), while the protein concentration was determined using the bicinchoninic acid (BCA) method (Cat. #PC0020, Solarbio, China). Equal amounts of protein were separated by SDS-PAGE. Subsequently, the gel containing the separated protein was transferred to a polyvinylidene difluoride membrane (PVDF, 0.2 µm pore size) membrane (Cat. No. #ISEQ00010, Millipore, USA). The PVDF membrane was then blocked with 10% low-fat milk for 30 min. Primary antibodies were diluted with primary antibody dilution buffer at the recommended concentration, and PVDF membranes were incubated in primary antibody solution overnight at 4 °C. After washing three times with TBST solution (20 min per time), the PVDF membranes were incubated together with the second antibody solution for 2 h at room temperature. Protein bands were then developed using an enhanced chemiluminescence kit (ECL) (Cat. No #32,106, ThermoFisher, USA) by the GV6000M developer (GelView 6000pro). Finally, the densitometric analysis of images was performed using ImageJ software [23], and protein expression was normalized with the that of GAPDH.

### Plasmid constructs and lentivirus packaging

Human PDIA4 overexpression plasmid, shRNA targets (shPDIA4-1: 5’-CCAAGAAGTACAAGGGCCAAA-3’; shPDIA4-2: 5’-GCAAGGTGTCAAACGATGCTA-3’; and shPDIA4-3: 5’-CCTGAGAGAAGATTACAAATT-3’) and 3Flag-tagged mutant PDIA4 (CXXC to CXXA) construct were designed and constructed by Genechem Company (Shanghai, China). The HA-tagged human VEGFA construct was designed and bought from Service company (Tianjing, China). The GV367 carrier (Ubi-MCS-SV40-EGFP-IRES-puromycin) was used to construct the PDIA4 overexpression and control plasmid. The GV248 construct (hU6-MCS-ubiquitin-EGFP-IRES-puromycin) was used to express the sh-RNAs. The GV141 construct (CMV-MCS-3FLAG-SV40-Neomycin) was used to express the 3Flag-tagged mutant PDIA4. The lentiviruses were also packaged with plasmids designed by Genechem Company (Shanghai, China).

### Sample preparation and RNA sequence analysis

Stable PDIA4 knockdown LN229 cells and the corresponding control cells were collected and diluted in TRIzol reagent (Cat. No #R0016, Beyotime, China) for RNA isolation, and RNA sequencing was performed by Nanjing Decode Genomics Company. Quality control was performed on the raw data. The raw data were transformed into reads per kilobase per million mapped reads (RPKM) format. Then, a panel of RNA-seq analyses was performed, including sample expression quantification, differential expression analysis (“limma” package) [24], and Metascape enrichment analysis (http://metascape.org/) [25] using a series of R packages and web tools.

### siRNA transfection

Two effective siRNAs targeting nonoverlapping sequences were selected from a previously published article [26]. The following scrambled siRNA and well-designed siRNAs targeting the XBP1 sequence were purchased from RiboBio (Guangzhou, China): XBP1 siRNA#1 sequence, 5’-CACCCUGAAUUCAUUGUCU-3’and XBP1 siRNA sequence # 2, 5'-CCAGGAGUUAAGACAGCGC-3'. The siRNAs were transfected into GBM cells using Lipofectamine 3000 (Cat. No. #L3000015, ThermoFisher, USA) in accordance with the manufacturer’s instructions. The effects of siRNAs were verified under the condition of tunicamycin (TM) -induced ER stress by western blotting. GBM cells were used to perform subsequent assays after 48 h of transfection.

### RNA extraction, reverse transcription polymerase chain reaction, and quantitative real-time PCR

Total RNA from GBM cells and samples was extracted using the RNA extraction kit (TIANGEN, Cat. No #DP419, China) according to the manufacturer’s instructions and the RNA concentration was quantified by N60-Touch (Implen, Germany). Then, 1ug of total RNA from each sample was used to synthesize complementary DNA (cDNA) and quantitative real-time PCR (RT-qPCR) was performed on the qTower3 G PCR system (Analytikjena, Germany) to quantify RNA expression levels. The sequences of primers used in this study are listed below: PDIA4 forward primer: 5’—TTGTTGGCGTAGATTTGGCT—3’, PDIA4 reverse primer: 5’—TGCTCAGTGGCAGCTCTCAC—3’; XBP1 forward primer: 5’—CGGTGCGTAGTCTGGAGCT—3’, XBP1 reverse primer: 5’—CCGACAGAAGCAGAACTTTAGG—3’; ATF6 forward primer: 5’—TAGCCCAGTGAATGGAAAACTT—3’, ATF6 reverse primer: 5’—CCTTAGCACAGCAATATCTGAACC—3’; GAPDH forward primer: 5’- GGTGAAGGTCGGAGTCAACG—3’, GAPDH reverse primer: 5’ – TGGGTGGAATCATATTGGAACA—3’.

### Immunohistochemical (IHC) staining

The clinical slices were reviewed by two experienced neuropathologists. Surgical tissues and GBM xenograft samples were fixed with 4% paraformaldehyde (PFA) and sectioned into 3-μm slices. Subsequently, the slides were deparaffinized, dehydrated, and incubated with 3% hydrogen peroxide in order. The slices were then boiled with 0.01 M sodium citrate antigen retrieval buffer (pH 6.0, Cat No #G1202, Servicebio, Wuhan, China). After blocking in 5% bovine serum albumin (BSA), primary antibodies were used at recommended concentrations to stain the slides overnight at 4 °C, followed by incubation with secondary antibodies (37 °C, 2 h). Proteins of interest were detected after DAB staining, and sections were finally counterstained with hematoxylin, and immunohistochemical (IHC) images were captured using a microscope (Leica, Wetzlar, Germany) and quantified using the ImageJ software “IHC Profiler” plugin [27]. The IHC score was defined as the equation:

$$IHC\;score\;\left(\%\right)=\frac{100-Percentage\;contribution\;of\;Negative}{100}\times100\%$$

### HUVEC migration assay

In the HUVEC migration assay, Transwell chambers (Cat. No. # 07–200-150, Corning, USA) with 8-μm pores in 24 well plates were soaked in the culture medium, and pre-heat in a 37 °C cell incubator for 4 h before adding cells. A total of 2 × 10^4^ HUVECs were added to the upper chamber wells containing serum-free DMEM. The same number of GBM cells for each group were pre-cultured in the 24-well plates, and then co-cultivated with HUVECs for 24 h in a 37 °C incubator. After removal of the cell culture medium, the migrating HUVEC cells were fixed with 4% PFA (Cat. No #P0099, Beyotime, China) for 30 min and stained with 0.25% crystal violet stain solution (Cat. No #G1061, Solarbio, Beijing, China) for 12 h. Images of migratory cells in the upper chambers were captured under an optical microscope (Leica), and the number of migrating HUVECs was calculated using ImageJ software.

### Tube formation assay

The reduced growth factor (GFR) Matrigel matrix (Cat. No. #354,263, Corning, USA) was diluted (1:1) using precooling cultured medium of each group of GBM cells on ice. Equably, we added 50 μL diluted Matrigel matrix in each well of a 96-well plate on the ice and bubbles were removed in each well. The 96-well plate was then placed in a 37 °C incubator for approximately 2 h to solidify the Matrigel matrix. Next, 50 μL conditioned medium containing 2 × 10^4^ HUVECs were added evenly to each well and incubated in a 37 °C incubator for 12 h before capturing images under optical microscope (Leica, Wetzlar, Germany). Tube numbers were calculated using the “Angiogenesis Analyser” plugin of Image J software [28].

### Enzyme-linked immunosorbent assay

Human VEGF levels secreted from each stable cell line were tested using an enzyme-linked immunosorbent assay (ELISA) kit purchased from Bioss Company (Cat. No. #bsk11024, Beijing, China). The testing procedure was performed in strict accordance with the manufacturer’s protocol.

### PDIA4 promoter binding prediction and chromatin immunoprecipitation assay

Open chromatin immunoprecipitation (ChIP) data in the Cistrome Data Browser (http://cistrome.org/db/#/) [29] were used to investigate the XBP-1 binding sites on the PDIA4 promoter, and visualization was performed using the website tool of the University of California Santa Cruz (UCSC) Genomics Institute (http://genome.ucsc.edu/). We used the Chromatin Immunoprecipitation Kit (Cat. No. #56,383, CST, USA) to perform ChIP assays. In the first step, 1% PFA was used to cross-link chromatin in tunicamycin-treated GBM cells, and subsequent procedures strictly followed the manufacturer’s instructions. Potential XBP-1 binding sites in the PDIA4 promoter sequence were predicted in advance in the JASPAR Core database (https://jaspar.genereg.net/) [30], and we selected the top 5 regions with the highest credibility to design the corresponding primers using the Primer Premier 6 software [31]. The immunoprecipitated DNA fragments were amplified using the following primer pairs, respectively: primer#1, 5ʹ-TTCTCCCACCACTGAGCAAATGʹ and 5ʹ-CACCGTGCCCAGCCTTAATATC-3ʹ; primer#2, 5ʹ-TGCTAAGGTCTGTGAGTTATCC-3ʹ and 5ʹ- TAACACCTGGGAGTTTGAGAAGʹ; primer#3, 5ʹ-CCTTCTCAAACTCCCAGGTGTT-3ʹ and 5ʹ-TTTCCCTCTTGACCACTTTGGA-3ʹ; primer#4, 5ʹ-CCACTAGATTGCCTATCTGGTA-3ʹ and 5ʹ-TCGGAGAAACAAGCCATCAG-3ʹ; and primer#5, 5ʹ-CAAACAGGCTCGTGCTCCTC-3ʹ and 5ʹ-CCGAACTTGGCAGTAAGAACAC-3ʹ. The PCR products were loaded into DNA blot and the XBP-1 binding DNA sequences were quantified and compared with the negative group.

### Dual-luciferase reporter assay

First, different promoter sequences of PDIA4, including wild full-length sequence, truncated mutated sequences and region 2 sequence, were cloned into the firefly-luciferase reporter plasmid, respectively. Together with the renilla-luciferase vector and siRNAs, they were co-transfected into U87 and LN229 cells. After 24 h of transfections, U87 and LN229 GBM cells were cultured with replaced medium containing 10 μg/mL of TM to induce ER stress. The luciferase activity was then quantified at 24 h after replacing the culture medium using a dual-luciferase reporter assay system (Cat. No #RG029M, Beyotime, Shanghai, China) according to the manufacturer’s instructions.

### Co-immunoprecipitation and mass spectrometry

The experimental steps were performed as described in a previous study [32]. First, U87 cells stably overexpressing the mPDIA4 flag were rinsed twice with PBS containing 20 mM N-ethylmaleimide (NEM, Cat. No #HY-D0843, MedChemExpress, USA), then lysed in NP40 cell lysis buffer (Cat. No #N8032, Solarbio, Beijing, China) containing 0.5 mM phenylmethylsulfonyl fluoride (PMSF, Cat No #36,978, ThermoFisher, USA). The total cell extracts were then centrifuged at 12,000 × g for 15 min at 4 °C and the supernatant was incubated with anti-Flag antibody overnight at 4 °C, then incubated with agarose beads (Cat. No #sc-2003, Santa Cruz) at 4 °C for 4 h, normal rabbit IgG as nonspecific control. Coimmunoprecipitation (Co-IP) lysates were prepared for LC-ESI- mass spectrometry (MS)/MS analysis to detect the substrate compositions, which was performed blindly by Micrometre Biotech Company (Hangzhou, China), or separated by SDS-PAGE for western blotting assay to validate the substrates. Normal IgG was also used to conduct the IP/MS analysis, and acted as a negative control to exclude false positive substrates.

### Intracranial xenograft mouse models

The animal experiments were approved by the NCUSAH Medical Ethics Committee following the UK Animals (Scientific Procedures) Act, 1986 and associated guidelines. Five-week-old BALB/c female nude mice (GemPharmatech, Nanjing, China) were used to establish intracranial xenograft GBM models. PDIA4 overexpressed and corresponding negative control U87 cells (with stable luciferase expression) were suspended in pre-cooled PBS, and 3 × 10^5^ U87 cells in 6 μL PBS were injected into the right frontal node of isoflurane anesthetized nude mice. The detailed superficial inoculation position was 2 mm lateral to the midline and 1 mm posterior to the bregma. Firstly, we drilled a hole with diameter of 1 mm, and inserted the needle vertically in 4 mm depth following exiting the needle 1 mm, then injected the GBM cells in 5 min. After injection, we keep the needle immobile for 5 min, when exiting the needle, we blocked the hole on the skull using bone wax. Tumor size was generally measured by luciferase intensity using the IVIS Lumina Series III system (Cat. No. 65391–72, PerkinElmer, USA). When the nude mice had abnormal activities or convulsions, they were executed using the cervical dislocation method. In glioma therapy experiments, temozolomide (TMZ) and bevacizumab (BEV) were administered at 40 mg/kg p.o. and 10 mg/kg i.v. respectively [33]. Brains were then removed and stored in 4% PFA for subsequently hematoxylin–eosin (HE) staining and immunohistochemical analysis.

### Statistical analysis

The paired t-test was used to compare the PDIA4 IHC score of the paired clinical GBM samples, and the t-tests were used to compare the levels of numerical variables between two experimental groups. The two-sided log-rank test was performed in the Kaplan–Meier model to compare the overall survival (OS) prognosis of patients with GBM in different groups. All statistical analyses in our investigation were performed using the R programming language (version 4.2.1, https://www.r-project.org/) and GraphPad Prism 9. Analysis with *p*-value < 0.05 were considered statistically significant.

## Results

### Up-regulated PDIA4 correlated with a poor prognosis for patients with GBM

**Fig. 1:**
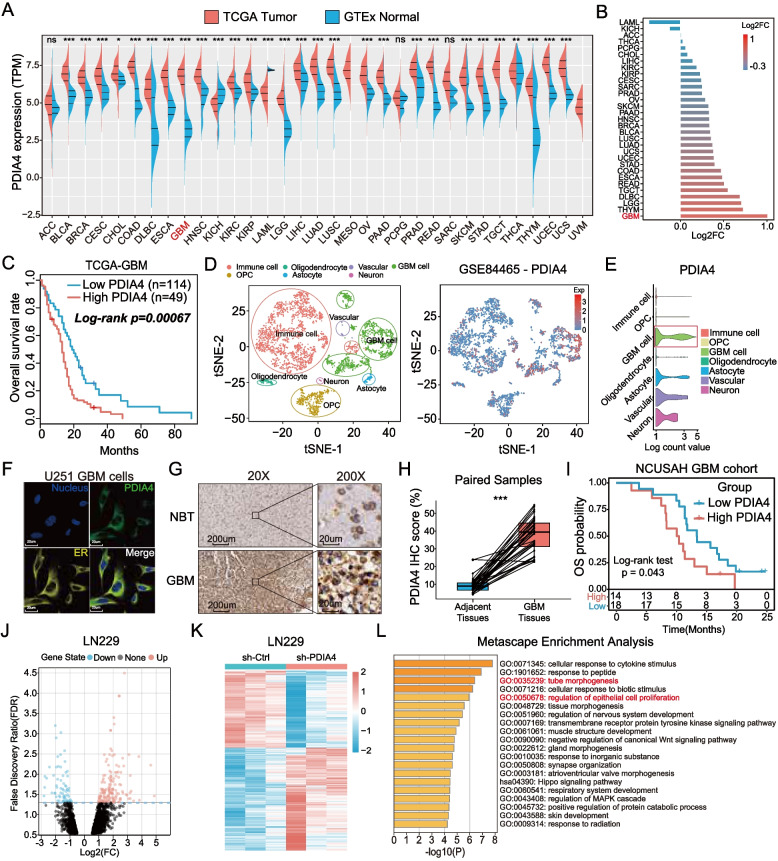
PDIA4 is extremely upregulated and correlates with worse prognosis of GBMs.** A-B** PDIA4 is overexpressed in 28 cancers, especially in GBM. ns *P* > 0.05; *P < 0.05; **P < 0.01; ***P < 0.001. **C** GBM patients with higher PDIA4 expression show poorer clinical prognosis in the TCGA cohort. **D** Scatter plots of the single-cell datset (GSE84465) showing the PDIA4 expressing distributions of different cell types in GBM microenvironment. **E** The violin plots of GSE84465 dataset showed that GBM cells expressed the highest PDIA4 in GBM microenvironment. **F** Immunofluorescence of U251 GBM cells indicates the subcellular localization in the ER of PDIA4 protein (Human Protein Atlas database). **G** Immunohistochemical staining of PDIA4 in clinical GBM samples and adjacent tissues showing distinct expression levels of PDIA4 protein. **H** Paired t-test was applied to compare the IHC scores of PDIA4 between adjacent tissues and GBM samples, and visualized in the box plots. ***P < 0.001. **I** The Kaplan–Meier survival analysis of GBM patients in neurosurgery department of NCUSAH verifies that higher PDIA4 expressions correlates worse clinical prognosis of GBM patients. **J** The volcano plot shows the RNA-seq analysis results of the different expressed genes (DEGs) between sh-Ctrl and sh-PDIA4 LN229 cells. **K** DEGs between sh-Ctrl and sh-PDIA4 LN229 cells were also visualized in this heatmap. **L** The Metascape enrichment analysis represents the gene ontology (GO) and KEGG pathway terms which these DEGs enriched in

As shown in Fig. 1A, PDIA4 was significantly upregulated in most cancers and was most significantly upregulated in GBM (Fig. 1B). Furthermore, the Kaplan–Meier prognosis analysis of four independent public cohorts of GBM revealed that patients with GBM with higher expression of PDIA4 were associated with a poorer OS prognosis (Fig. 1C, Figure S1A-C). These data indicated that abnormally upregulated PDIA4 probably play a vital role in the malignant progression of GBM. Then, we investigated which cell type was primarily responsible for expressing PDIA4 in the GBM microenvironment. The single cell RNA-seq data (scRNA-seq) from the GSE84465 dataset, including 3589 cells from four GBM samples, were downloaded and analyzed. Based on the results of the scRNA-seq analysis, we determined that PDIA4 was expressed primarily in GBM cells (Fig. 1D, E). Immunofluorescent images of the Human Protein Atlas (HPA) data set have shown that PDIA4 is a protein localized in the endoplasmic reticulum (ER) (Fig. 1F), which is consistent with the protein folding and secretion functions of PDIA4 in the ER. The IHC assay labelled PDIA4 in 32 paired clinical samples and verified that PDIA4 was overexpressed in GBM samples compared to adjacent tissues (Fig. 1G). The paired comparison analysis showed higher PDIA4 IHC scores in GBM samples (Fig. 1H). By combining follow-up data and quantified IHC scores, patients with GBM were classified from high expression of PDIA4 to low, and Kaplan–Meier analysis also confirmed that clinical GBM patients with higher protein expression of PDIA4 had a shorter survival period and lower survival rate (Fig. 1I).

Based on current results, we speculated that PDIA4 might be involved in the malignant progression of GBM. Thus, we obtained PDIA4 overexpression and knockdown lentiviral vectors for subsequent in vitro experiments. First, we tested the levels of PDIA4 protein in five GBM cell lines compared to human astrocyte (HA) cells, most GBM cells express higher PDIA4 than HA cells. Among the GBM cell lines, LN229 had highest expression of PDIA4, while U87 showed lowest expression (Figure S1D), thus we designed our subsequent experiments using PDIA4-knockdown (sh-PDIA4) LN229 cells and PDIA4-overexpressing (LV-PDIA4) U87 cells. The knockdown and overexpression results were verified by western blotting (Figure S1E). The most significant knockdown shRNA was chosen for subsequent RNA sequencing analysis in LN229 cells to identify the potential functions of PDIA4 in GBM malignancy.

### RNA-seq analysis revealed the downstream effects of PDIA4 on the transcriptomic level in GBM cells

To better understand the role of PDIA4 in the progression of GBM cells, we knocked down PDIA4 expression in LN229 cells using sh-PDIA4-3. The sh-PDIA4-3 and sh-Ctrl LN229 cells were collected to perform whole transcriptome sequencing analysis. Subsequently, differential expression analysis between sh-PDIA4-3 and sh-Ctrl LN229 cells identified 241 differential expression genes (False Discovery Ratio, FDR < 0.05), these DEGs were visualized using a volcano plot and heatmap (Fig. 1J and K). Gene enrichment analyses, including GO-BP and KEGG pathway analyses, were performed using the Metascape webtool [25] and the results are presented in Fig. 1L. At the same time, similar analyses using TCGA-GBM transcriptomic data were performed, and the related Metascape enrichment results are presented in Figure S2A. The top ten enriched terms of GO biological processes (GO-BP) or KEGG pathways are represented in the bar plots, respectively. According to the RNA sequence analysis of PDIA4 knockdown GBM cells, we observed that PDIA4 positive genes were associated with biological processes or pathways such as the cellular response to cytokine stimulus, response to peptide, tube morphogenesis, and regulation of epithelial cell proliferation (Fig. 1L). The same analysis in the TCGA-GBM cohort indicated that PDIA4 was involved in protein folding in the ER, activation of NF-κB transcription factors, and the positive regulation of cytokine production (Figure S2A).

### Identification of VEGFA as the substrate of PDIA4

**Fig. 2:**
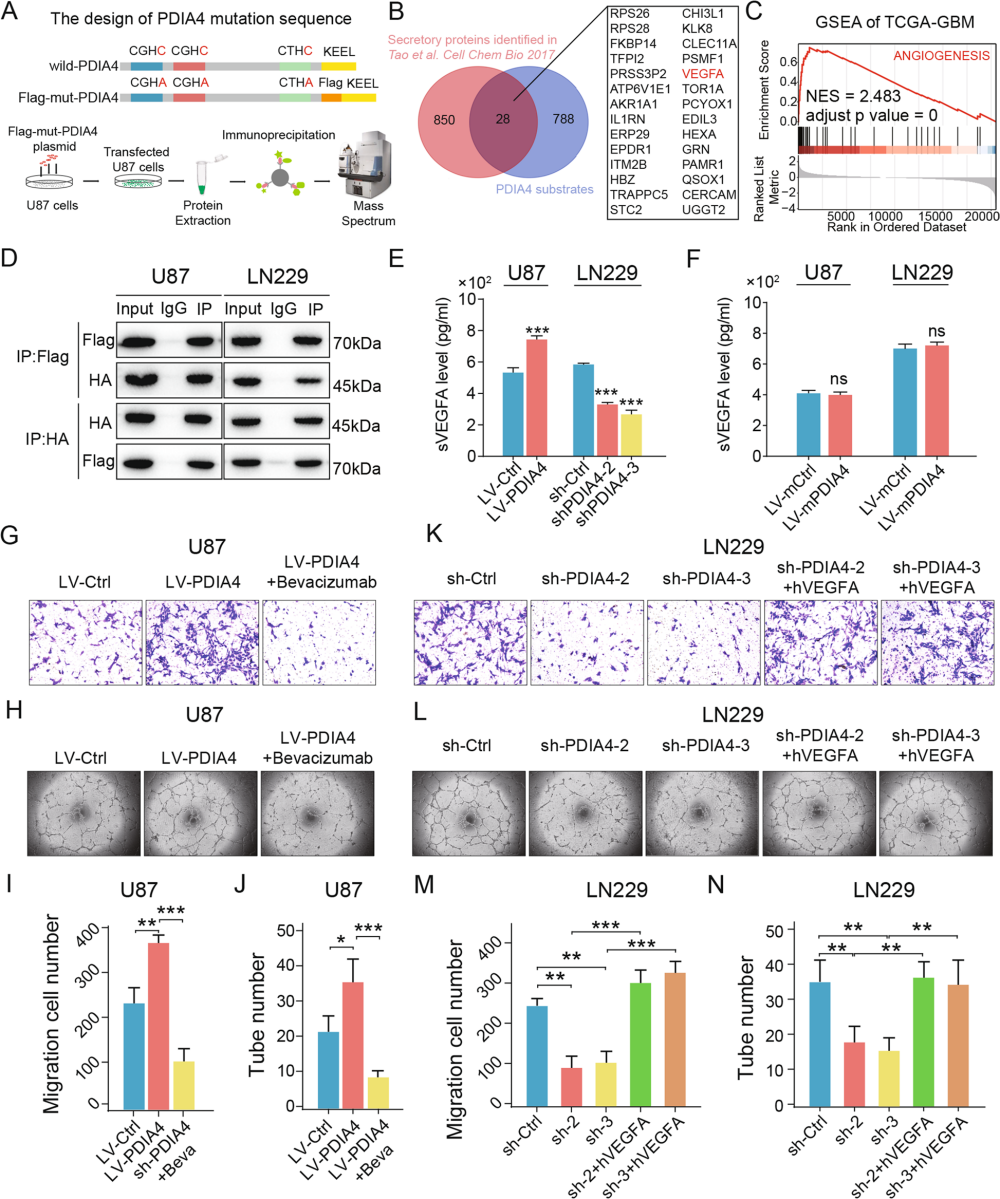
Identification of secretory protein substrates of PDIA4 in GBM cells. **A** The general view of the construction of PDIA4 mutant plasmids, co-immunoprecipitation assay, and mass spectrum (MS) identification of PDIA4 potential substrates in U87 cells. **B** The Venn diagram shows twenty-eight proteins were identified as the secretary substrates of PDIA4 in GBM by intersecting 816 PDIA4 substrates and 878 human secretory proteins identified in *Tao *et al*. Cell Chem Bio 2017*. **C** Gene set enrichment analysis (GSEA) indicates the hallmark of angiogenesis is significantly enriched in high-PDIA4 GBM subgroup in the TCGA-GBM cohort. **D** Co-immunoprecipitation and western blot assays show the direct interaction between Flag-mPDIA4 and HA-VEGFA in U87 and LN229 GBM cells. **E** PDIA4 upregulation in U87 cells increases while knock-down of PDIA4 in LN229 cells decreases the VEGFA secretion in ELISA assay. ***P < 0.001. **F** Overexpression of mPDIA4 in U87 and LN229 GBM cells didn’t influence VEGFA secretion. ns *P* > 0.05. **G-J** Transwell migratory and tube formation assays of co-cultured HUVECs shows upregulation of PDIA4 in U87 cells can promote HUVEC migratory and tube formation capacities, which can be blocked by bevacizumab. *P < 0.05; **P < 0.01; ***P < 0.001 **K-N** Transwell migratory and tube formation assays of co-cultured HUVECs shows downregulation of PDIA4 in LN229 cells can decrease HUVEC migratory and tube formation capacities, which can be rescued by additional exogenous human VEGFA. **P < 0.01; ***P < 0.001

To identify downstream substrates of PDIA4 in GBM cells, we referred to the methods used in previous publications [32, 34], and create stable GBM cell lines expressing the mutant version of PDIA4 containing three mutations from CXXC to Cys-X-X-Ala (CXXA) at the three active sites with the flag tag before the C-terminal KEEL sequence (Fig. 2A). Mutations in the latter cysteines at the active sites could prolong the binding time of the enzyme reaction to PDIA4 substrates, and is a commonly used approach to capture the substrates of PDIs [32, 34]. When the Co-IP and protein MS analyses were combined, the peptide fragments of the substrates were identified. To obtain more convincing results, normal rabbit IgG (negative control of anti-flag IgG) was used to perform the same procedure as the negative control. The final results were obtained by subtracting the identified substrates of anti-Flag from those of the anti-IgG negative control.

Considering the potential role of PDIs in regulating protein maturation and secretion [5, 35, 36], we focused on secretory proteins in identified substrates. According to the work of Tao et al. (2017) [37] in identifying secretory proteins, we obtained a list of gene names of a total of 878 proteins secreted from human cells (Supplementary Table 1). By intersecting with the 816 PDIA4 substrates (Supplementary Table 2), we obtained 28 potential secretory substrates of PDIA4, the Venn diagram showed the intersection procedure and 28 secretory PDIA4 substrates (Fig. 2B).

Based on the enrichment results of tube morphogenesis and regulation of epithelial cell proliferation (shown in Fig. 1L, in red) in the RNA-seq analysis, the VEGFA, as one of the potential substrates of PDIA4, attracts our eyes. The FMDVYQR peptide of human VEGFA was identified in these substrates (Figure S2B), thus we speculated that PDIA4 could play a role in GBM angiogenesis by regulating VEGF secretion. We then validated the association between PDIA4 expression and the hallmark of angiogenesis in the TCGA-GBM cohort by gene set enrichment analysis (GSEA), and the results showed that the hallmark of angiogenesis was significantly enriched in high expression GBM of PDIA4 (Fig. 2C, NES = 2.483, adjusted *p* value < 0.0001).

Next, we verified the intracellular interaction between PDIA4 and VEGFA by protein co-IP and western blot assays. The VEGFA-HA overexpression plasmid was transfected into LV-mPDIA4 U87 and LN229 cells, respectively. Then co-IP combined with western blotting were used to validate the direct interaction between them in GBM cells. Immunoblot bands indicated that there was a robust interaction between PDIA4 and VEGFA intracellularly (Fig. 2D). This evidence indicated that PDIA4 might regulate GBM angiogenesis by promoting the folding and secretion of the VEGFA protein.

### PDIA4 accelerated GBM cell derived VEGFA secretion

To investigate whether PDIA4 regulates VEGFA secretion in GBM cells, we detected the expression and secretion of VEGFA protein in GBM cells with different expressions of PDIA4, by immunoblotting and ELISA assays. Intracellular VEGFA expression was up-regulated in LV-PDIA4 compared with LV-Ctrl U87 cells, but down-regulated in sh-PDIA4 compared with sh-Ctrl LN229 cells (Figure S2C). Furthermore, GBM cells with different expression of PDIA4 also showed different levels of secretion of VEGFA. By performing ELISA assay, we determined LV-PDIA4 U87 cells secreted more VEGFA in vitro culture medium, and sh-PDIA4 LN229 cells secreted less VEGFA (Fig. 2E). Next, 48 h after transfected, we detected secretion of VEGFA in LV-mPDIA4 and LV-mCtrl U87 and LN229 cells by ELISA, but no significant changes in VEGFA secretion were observed (Fig. 2F). Therefore, these findings confirmed that PDIA4 regulates VEGFA secretion through its disulfide isomerase activity.

### Endogenous PDIA4 levels in GBM-controlled co-cultured HUVEC migration and tube formation in vitro

To evaluate the bioactivity of secreted VEGFA regulated by PDIA4, we used the Transwell co-culture system to access the migratory capacity of HUVEC cells, and the GBM conditioned medium was used to perform the tube formation assay. HUVEC co-cultured with LV-PDIA4 U87 cells showed higher migratory and tube formation abilities in vitro, and this enhancement could be blocked by adding bevacizumab (BEV, a VEGFA-specific antibody) (Fig. 2G-J). Furthermore, the elimination of PDIA4 in LN229 cells decreased the migratory and tube formation abilities of co-cultured HUVEC and the addition of exogenous human-VEGFA (hVEGFA) could completely rescue these deficits (Fig. 2K-N).

### ER stress induced PDIA4 expression through transcriptional regulation of XBP-1

As previously reported, PDIA4 is an ER stress chaperonin and is up-regulated under ER stress [38]. However, its underlying mechanisms and its main regulator in GBM cells are unclear. To clarify this problem, we constructed an ER stress GBM-induced cell model using TM (10 μM/mL), which is an ER stress inducer by inhibiting N-linked glycosylation and blocking GlcNAc phosphotransferase (GPT), resulting accumulation of in unfolded protein in ER [39, 40].

First, we chose XBP1, ATF6, and HIF1A as candidate transcriptional factors (TFs) according to a previous publication [38] and public data analysis. All three TFs are upregulated in GBM compared to normal brain tissues (Figure S3A) and showed strong expression correlations with PDIA4 (Figure S3B). GSEA also indicated that GBM with higher PDIA4 is significantly enriched with hypoxia hallmark (Figure S3C). We then searched publicly available ChIP data and it showed that HIF1α strongly bound to the promoter region of PDIA4 in T47D cells, but did not in PC-3 cells (Figure S3D). Therefore, hypoxia-treated experiments were performed with GBM cells and immunoblotting was used to detect changes in expression. We did not observe any upregulation in PDIA4 expression under 0% oxygen treatment after 6, 12, 24 h of culture (Figure S3E). These data excluded the assumption that hypoxia induced PDIA4 expression in GBM cells.

**Fig. 3:**
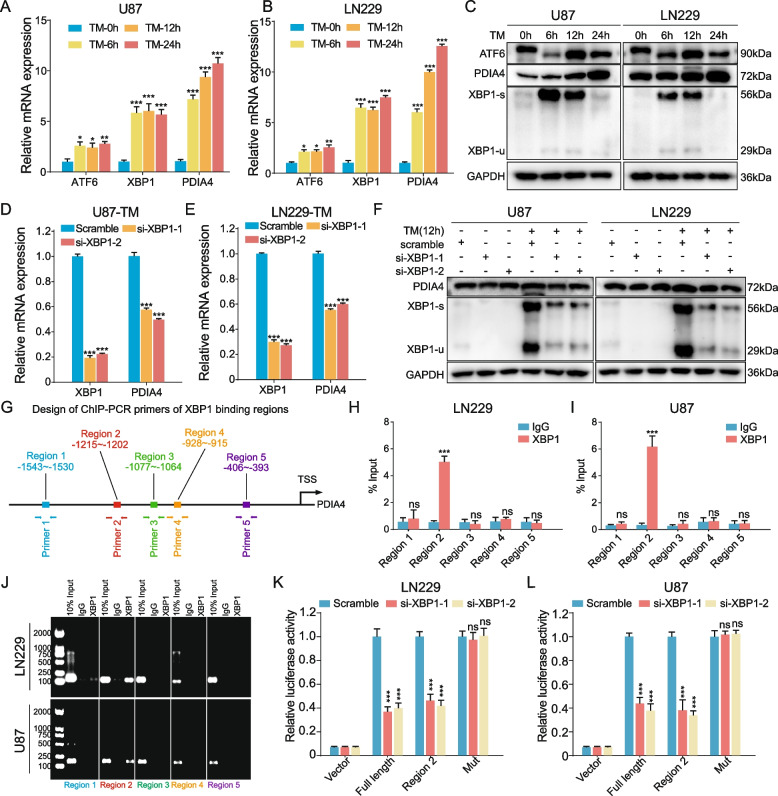
ER stress induced XBP1 upregulates PDIA4 expression transcriptionally. **A-B** RT-qPCR quantified the relative mRNA expressions of PDIA4, XBP1 and ATF6 in TM-induced ER stressed U87 (**A**) and LN229 (**B**) cells at 0, 6, 12 and 24 h. *P < 0.05; **P < 0.01; ***P < 0.001. **C** Western blot assay showed the ATF6, XBP1-s, XBP1-u and PDIA4 protein expressions were all upregulated under TM-induced ER stress in GBM cells. **D-E** Blocking XBP1 in ER stressed GBM cells by siRNAs significantly downregulates PDIA4 mRNA expressions. ***P < 0.001. **F** Upregulation of PDIA4 protein under ER stress was intercepted by blocking XBP1 expressions in GBM cells. **G** Design of ChIP-PCR primers of XBP1 binding regions. **H-I** The ChIP-PCR results showed Region 2 sequence (-1215 ~ -1202) on PDIA4 promoter were captured by XBP1 protein immunoprecipitation in U87 (**H**) and LN229 (**I**) cells. ns *P* > 0.05; ***P < 0.001. **J** The DNA gel electrophoresis shows the abundance of PCR produced DNA sequences of ChIP assay. **K** The luciferase activities of the vector, full length, region 2 and mutant PDIA4 promoter sequence transfected LN229 cells with or without XBP1 knock-down under 10 μg/mL TM induced ER stress. ns *P* > 0.05; ***P < 0.001. **L** The luciferase activities of the vector, full length, region 2 and mutant PDIA4 promoter sequence transfected U87 cells with or without XBP1 knock-down under 10 μg/mL TM induced ER stress. ns *P* > 0.05; ***P < 0.001

We then treated U87 and LN229 cells with 10 μM/mL of TM to induce ER stress and then detected the expression of XBP1, ATF6, and PDIA4 mRNA and proteins at the time points of 0 h, 6 h, 12 h, and 24 h. The mRNA and protein expression of XBP1, ATF6, and PDIA4 all significantly increased under induction of ER stress according to the RT-qPCR (Fig. 3A, B) and immunoblotting (Fig. 3C) results. However, when used Jaspar webtool to predict potential ChIP outcomes, there was no evidence of a binding domain of ATF6 in the promoter region of PDIA4. Besides, the public ChIP-seq analysis showed there are significant XBP1 binding sites on PDIA4 promoter in MDA-MB-231, T47D and HS578T cells (Fig. S3F), we then selected XBP1 to continue our analysis. Two siRNA targeting sequences of human XBP1 were obtained from a previous study [26], and were transfected into U87 and LN229 cells. We then treated these GBM cells with 10 μM/mL TM to induce ER stress; related control groups were also included in the experimental design. RT-qPCR assays showed that PDIA4 mRNA expression was negatively regulated, while XBP1 expression was blocked under stress conditions in the ER in both U87 (Fig. 3D) and LN229 cells compared to untreated control cells (Fig. 3E). Typically, PDIA4 protein expression was also reduced when XBP1 expression was blocked by siRNAs under ER stress (Fig. 3F). Therefore, XBP1 was targeted as a candidate transcriptional regulator of PDIA4 under ER stress conditions.

Based on the results of the Jaspar webtool prediction, we designed primers targeting the five regions with the highest binding potential (Fig. 3G) for the subsequent ChIP assay. The RT-qPCR results indicated that Region 2 (-1214 ~ -1202) in the PDIA4 promoter sequence was the most convincing motif for the binding of XBP1 in GBM cells (Fig. 3H-I). While the DNA gel blot visually represented the enriched binding of XBP1 in Region 2 in GBM cells (Fig. 3J). Furthermore, we also designed a dual-luciferase reporter assay to validate the direct interaction of the XBP1 protein and the Region 2 sequence on the PDIA4 promoter. The full-length promoter sequence (-2000 to -1 bp) sequence of PDIA4, the clipped forms of the PDIA4 promoter including region 2 (-1150 to -1250 bp), and the full-length promoter sequence of PDIA4 with region 2 mutation were cloned into the vector plasmid, respectively. The assays were conducted under stress conditions (10 μM/mL) to induce the expression of XBP1 in GBM cells. These results suggested that silencing ER stress-induced XBP1 inhibited the transcriptional activity of the PDIA4 promoter, but not that of the region 2 mutation sequence (Fig. 3K-L). Overall, these findings indicated that region 2 of the PDIA4 promoter could be the only binding site for XBP1. In conclusion, these data confirmed that PDIA4 is transcriptionally upregulated by XBP1 under ER stress conditions.

### ER stress induced GBM-derived VEGFA secretion depending on the XBP1/PDIA4 axis

**Fig. 4:**
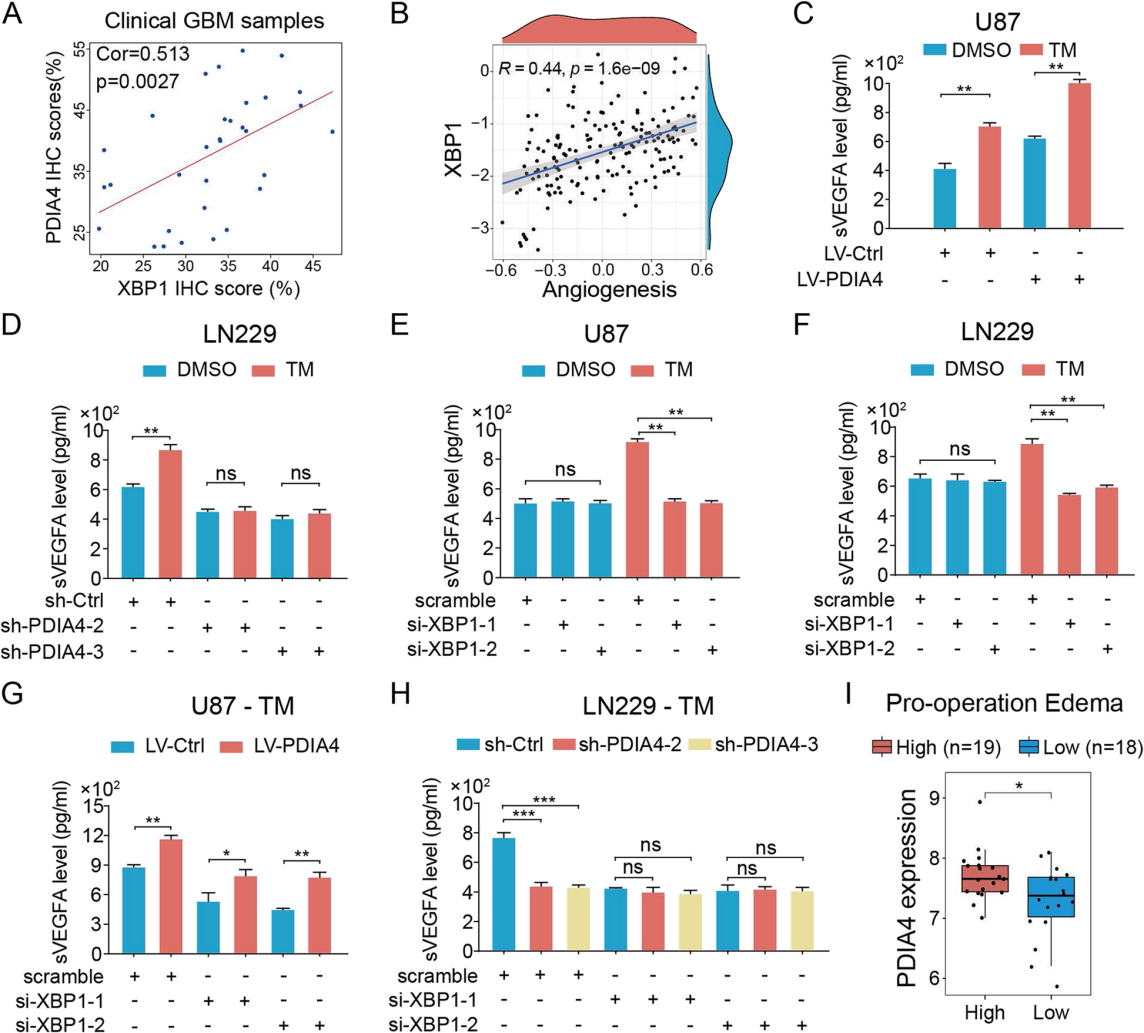
ER-stress induces XBP1/PDIA4/VEGFA regulatory axis in GBM. **A** The Pearson correlation analysis between XBP1 and PDIA4 IHC scores of clinical GBM samples reveals a strong positive correlation. **B** The Pearson correlation analysis between XBP1 expression and GSVA score of angiogenesis hallmark of GBM samples in the TCGA cohort. **C** The VEGFA secretion levels of LV-Ctrl and LV-PDIA4 U87 GBM cells with or without 10 μg/mL TM treatment. **P < 0.01. **D** The VEGFA secretion levels of sh-Ctrl and sh-PDIA4 LN229 GBM cells with or without 10 μg/mL TM treatment. ns *P* > 0.05; **P < 0.01. **E–F** The VEGFA secretion levels of XBP1 knock-down U87 (**E**) and LN229 (**F**) cells with or without 10 μg/mL TM treatment. ns *P* > 0.05; **P < 0.01. **G** The VEGFA secretion levels of XBP1 knock-down in LV-Ctrl and LV-PDIA4 U87 cells under 10 μg/mL TM induced ER stress. *P < 0.05; **P < 0.01. **H** The VEGFA secretion levels of XBP1 knock-down in sh-Ctrl and sh-PDIA4 LN229 cells under 10 μg/mL TM induced ER stress. ns *P* > 0.05; ***P < 0.001. **I** PDIA4 expression in high pro-operation edema GBMs is higher than low pro-operation edema GBMs. *P < 0.05

We then aimed to explore the function of XBP1/PDIA4/VEGFA axis in GBM cells under ER stress. Pearson’s correlation analysis between the IHC scores of XBP1 and PDIA4 showed that a strong positive correlation existed between the expression of the XBP1 and PDIA4 proteins in clinical GBM **(**Fig. 4A). Bioinformatics analysis suggested a strong correlation between the GSVA score for angiogenesis and the expression of XBP1 in the TCGA-GBM cohort (Fig. 4B).

Subsequently, we investigated the role of the regulatory axis XBP1/PDIA4 in ER stress-induced angiogenesis in vitro. It is indicated that TM-induced ER stress could enhance VEGFA secretion in both LV-PDIA4 and LV- Ctrl U87 cells (Fig. 4C). However, TM-induced ER stress cannot increase VEGFA secretion in sh-PDIA4 LN229 cells but sh-Ctrl cells (Fig. 4D). These findings suggested that blocking PDIA4 can inhibit the pro-angiogenesis ability of GBM cells under ER-stress condition, which is not good for GBM cell surviving in tumor microenvironment. Furthermore, to verify the role of XBP1 in ER stress induced VEGFA secretion, we then inhibited XBP1 expression using siRNAs in GBM cells under different stress conditions. In GBM cells without ER stress, knockdown of XBP1 did not influence VEGFA secretion (Fig. 4E, F, blue bar plots), this is because XBP1 is rarely expressed in normally cultured GBM cells without ER-stress in vitro (Fig. 3F), but it significantly blocked ER stress induced VEGFA secretion of GBM cells (Fig. 4E, F, red bar plots). To determine if the XBP1/PDIA4 regulation plays a vital role in ER-induced VEGFA secretion, we knock-down XBP1 in U87 and LN229 cells with different PDIA4 expressions under ER-stress. We found that overexpression PDIA4 can rescue the decrease of VEGFA secretion of U87 cells while XBP1 is inhibited under ER-stress condition (Fig. 4G). Oppositely, knock-down of PDIA4 or not in LN229 cells with XBP1 inhibition under ER-stress showed no significance in VEGFA secretion (Fig. 4H). This data indicates that XBP1/PDIA4 axis plays a crucial role in ER-stress induced angiogenesis by improve VEGFA secretion.

Considering the effect of PDIA4 in regulating VEGFA secretion of GBM cells, we supposed that PDIA4 expression is associated with peritumoral edema of GBMs. By obtaining imaging data from TCGA-GBM patients, we analyzed PDIA4 expression across GBM subgroups with different levels of pre-operative edema (prop-edema) [41]. Interestingly, we found that PDIA4 was highly expressed in GBM patients with high prop-edema (Fig. 4I). This result drove us to investigate if PDIA4 expression was associated with antiangiogenic therapy of GBM patients.

### PDIA4 facilitated GBM growth, poor prognosis, and angiogenesis in vivo

**Fig. 5:**
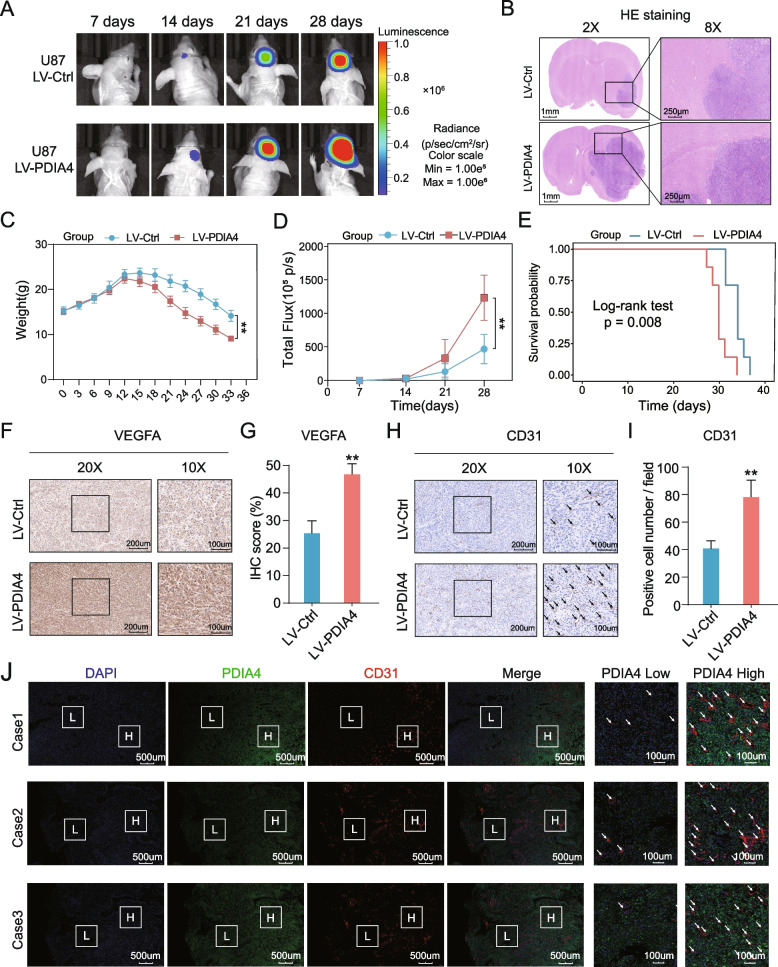
PDIA4 facilitated GBM growth, poor prognosis, and angiogenesis in vivo. **A** In vivo xenograft U87 GBM tumors were imaged at 7, 14, 21, and 28 days after the intracranial tumor was implanted using the IVIS system. **B** Representative HE staining images show the distinct size of xenograft U87 GBM with different PDIA4 expression. **C** The line chart shows the weight variation of GBM-bearing nude mice in two groups. **P < 0.01. **D** Recorded total flux at each time point indicates growing xenograft tumor size of each nude mice group dynamically. **P < 0.01. **E** Kaplan–Meier survival analysis showed nude mice with overexpression PDIA4 xenograft had poorer outcomes. **F** Representative VEGFA IHC images in two mice groups. **G** IHC scores of VEGFA in high-PDIA4 xenograft GBMs are significantly higher than low-PDIA4 xenograft GBMs. **P < 0.01. **H** Representative CD31 IHC images in two mice groups.** I** CD31-positive cell number in high-PDIA4 xenograft GBMs are significantly higher than low-PDIA4 xenograft GBMs. **P < 0.01. **J** Immunofluorescence of PDIA4 (green), CD31 (red) and DAPI (blue) in three cases of GBM samples

To clarify the malignant role of PDIA4 in the progression of GBM in vivo, luciferase labelled LV-PDIA4 and LV-Ctrl U87 cells were injected into the brains of two groups of nude mice, respectively. We chose U87 rather than LN229 cells to establish the GBM xenograft model because of the higher tumor formation rate of U87 cells in nude mice. In vivo tumors were imaged at 7, 14, 21, and 28 days after the intracranial tumor was implanted (Fig. 5A) using the IVIS system, and the survival time was recorded, mice brains were harvested after the mice died. Representative HE staining images were used to show differences in tumor size between the two xenograft tumor groups (Fig. 5B). The body weights of all mice were recorded every three days after tumor implants, and the line graphs indicated that PDIA4 overexpression tumors conferred mice a more rapid reduction in body weight starting from approximately 15 days to the endpoint (Fig. 5C). Periodical flux monitoring demonstrated that xenograft tumors with LV-PDIA4 exhibited higher total flux compared to the LV-Ctrl, indicating that PDIA4 drives greater progressive tumor growth in vivo (Fig. 5D). IHC analysis of xenograft tumors indicated stronger staining of VEGFA and CD31 in LV-PDIA4 xenograft tumors (Fig. 5F-I), which indicated tumor-derived PDIA4 could promote VEGFA secretion and angiogenesis in vivo, as we had speculated. We then performed co-immunofluorescent staining of PDIA4 and CD31 in three clinical GBM samples. All three cases showed that intratumor regions with higher expression of PDIA4 were enriched with more CD31 staining (Fig. 5J), which indicated greater enrichment of blood vessels in regions with higher expression of PDIA4.

### Higher PDIA4-expressing GBMs exhibited greater resistance to antiangiogenic therapy

**Fig. 6:**
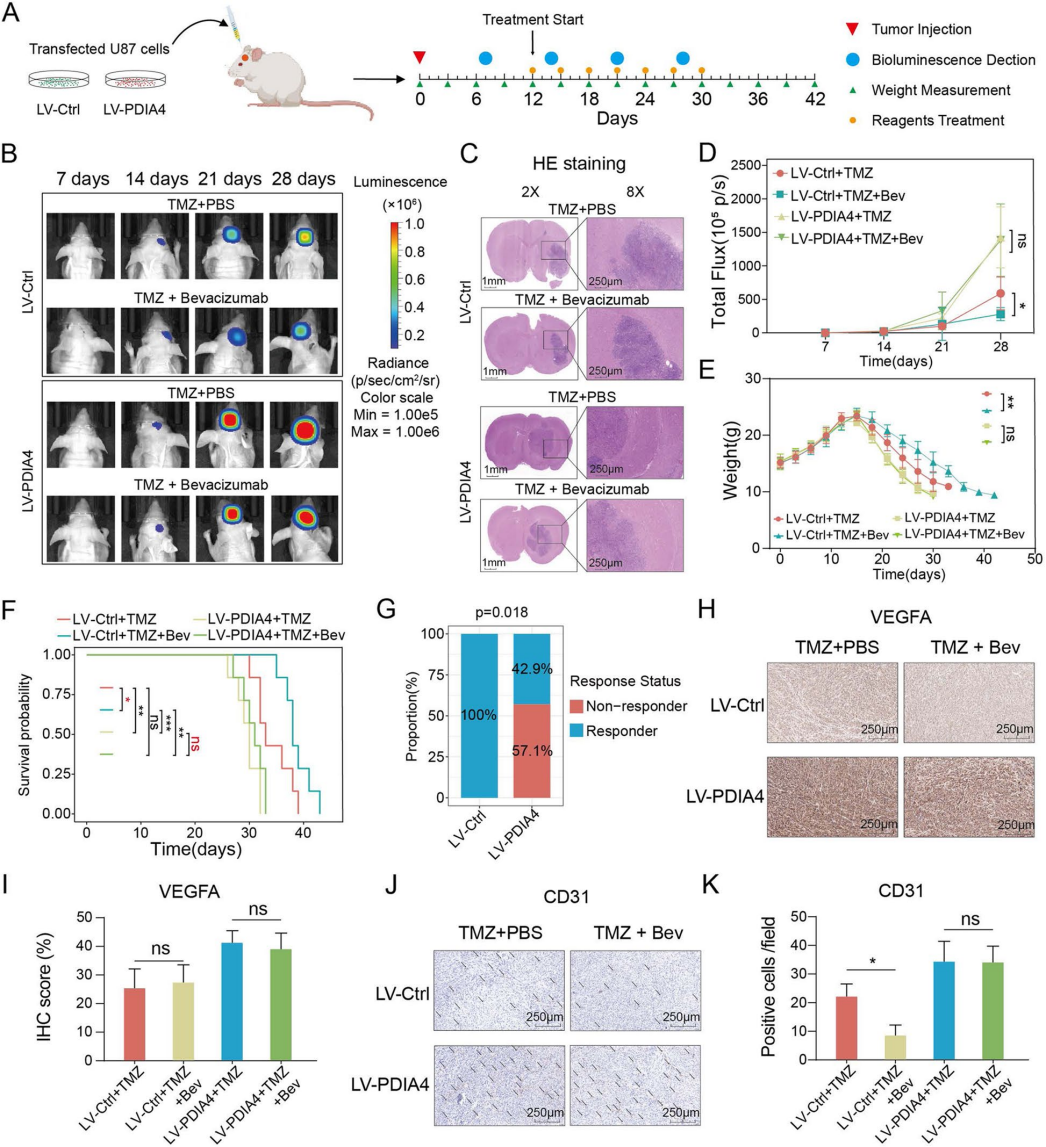
PDIA4 confers glioblastoma resistance to anti-angiogenic therapy. **A** The workflow of animal therapy study. **B** In vivo GBMs were imaged at 7, 14, 21, and 28 days after the intracranial tumor was implanted using the IVIS system. **C** Representative HE staining images show the tumor size of xenograft GBM in each group. **D** Recorded total flux at each time point present tumor size of each nude mice group dynamically. ns *P* > 0.05; *P < 0.05. **E** The line chart exhibits the weight variation of nude mice in each group. ns *P* > 0.05; **P < 0.01. **F** Kaplan–Meier survival analysis showed the prognosis of each mice group. ns *P* > 0.05; *P < 0.05; **P < 0.01; ***P < 0.001. **G** The number of anti-angiogenic therapy responder in each group were compared (Chi-square test, *p* = 0.018). **H** Representative VEGFA IHC images of each xenograft GBM mice group with different treatment. **I** Bar plots show the IHC scores of VEGFA in each xenograft GBM mice group with different treatment. ns *P* > 0.05. **J** Representative CD31 IHC image of each xenograft GBM mice group with different treatment. **K** Bar plots show the CD31 + cell number in each xenograft GBM mice group with different treatment. ns *P* > 0.05; *P < 0.05

We speculated that PDIA4 could be a clinical therapeutic biomarker for patients with GBM. Thus, we investigated the potential clinical implications of PDIA4 in the response to antiangiogenic therapy of GBM patients. We designed four groups of nude mice in which two groups were implanted with LV-PDIA4 cells and another two groups were injected with LV-Ctrl U87 cells. Each subgroup of mice was treated with TMZ with or without BEV to verify the potential role of PDIA4 on antiangiogenic therapy of GBM. A schematic diagram of the study flow of the animal experiment is shown in Fig. 6A. Representative bioluminescence images revealed the dynamic growth of GBM in each group (Fig. 6B), and HE staining indicate the representative tumor size at the end points (Fig. 6C). Based on the weight, flux intensity, and survival data we collected, we visualized the changes in flux intensity, body weight, and survival probabilities of each group. The total flux intensity was used to indicate the tumor size of each mouse. TMZ and BEV combination therapy showed a significant suppressive effect of GBM in the LV-Ctrl GBMs, but lost efficacy in GBM with higher expression of PDIA4 (Fig. 6D). The dynamic body weight variant curves showed that the mice in the LV-PDIA4 group decreased rapidly compared to the LV-Ctrl groups, and no significance changes were found between TMZ and TMZ plus Bev therapy in LV-PDIA4 mice. However, the combination therapy showed significant efficacy for the LV-Ctrl group of mice (Fig. 6E). The survival curves also support the above findings: combination therapy achieved a more curative effect in LV-Ctrl GBMs, which ultimately lead to a prolonged survival of LV-Ctrl GBM-bearing mice (Fig. 6F). To better describe our results, we defined nude mice responded to antiangiogenetic therapy and survived longer than the median survival time of their single TMZ-treated counterparts and were defined as “antiangiogenetic therapy responsive”, with the mice exhibiting shorter survival defined as “nonresponsive”. The results revealed that there were more non-responders in GBM-bearing mice with higher expression of PDIA4 than in mice with lower PDIA4 (Fig. 6G). The immunohistochemical staining of VEGFA and CD31 in U87 xenograft tumors from each group was then performed. The results indicated that BEV therapy did not influence VEGFA in GBM or in its microenvironment as no significant differences were observed between TMZ monotherapy and TMZ and BEV combination therapy in both the GBM subgroups LV-Ctrl and LV-PDIA4 (Fig. 6H-I). However, CD31 staining differed between the two therapy methods: compared to the LV-Ctrl GBM subgroup, and no significance differences were observed in the LV-PDIA4 group (Fig. 6J-K), which further demonstrated that antiangiogenetic therapy was effective in GBM with lower expression of PDIA4 than in GBM with higher expression of PDIA4.

## Discussion

Tumor cells survive in a severe microenvironment characterized by hypoxia, low pH, and hypoglycemia due to the growth of uncontrolled oncocytes combined with unrestricted neovascularization. These extrinsic ER stressors induce intracellular protein production and increased folding, so that tumor cells adapt their survival strategy by activating the UPR pathway [42, 43]. Multiple studies have focused on investigating how cancer cells survive in harsh microenvironments, which might illustrate how the growth potential of cancer cells during carcinogenesis and progression can be targeted, but these complexes require need further investigation.

In our study, PDIA4 was markedly upregulated in clinical GBMs and indicates a poor prognosis. PDIA4 also enhances the angiogenesis capacity of GBM cells in vitro and in vivo*,* by its oxidoreductase activities in the regulation of the expression and secretion of the GBM-derived VEGFA protein. As a protein chaperon and folder, it also participates in the UPR pathway activated by ER stress, transcriptionally regulated by XBP-1. Furthermore, the XBP1/PDIA4/VEGFA regulatory axis plays a vital role in ER stress-induced angiogenesis and confers resistance of the GBM to antiangiogenetic therapy in GBM xenograft nude mice. These results provide a novel mechanism to GBM cell survival in a harsh microenvironment by regulating the XBP1/PDIA4/VEGFA pathway, which helps clarify the role of the UPR pathway in the progression of GBM. Furthermore, we identified an association between PDIA4 and the response to antiangiogenetic therapy in patients with GBM, this finding could be meaningful in terms of its impact on the targeted treatment of patients with GBM.

Evidence is emerging that supports bevacizumab, an FDA-approved antiangiogenetic drug, as a promising drug in combination chemotherapy strategies to improve the PFS of patients experiencing GBM recurrence, although none have improved OS in these patients [44–46]. Our study reveals that PDIA4 confers greater resistance of GBM to TMZ/BEV combination therapy due to its pro-angiogenesis effects on GBM. Meanwhile, PDIA4 could act as a clinically useful indicator to guide precision antiangiogenetic therapy in patients with GBM. The human PDIA4 coding sequence is located on chr7q, which is one of the most frequent amplified chromosomes in patients with GBM [47], this clarifies its overexpression in GBM, and could explain the resistance to antiangiogenetic therapy in patients with GBM.

Multiple lines of evidence have proved that the HIF1α pathway is vital for glioma angiogenesis [48], and sensors associated with IRE1α and XBP1 have been reported to be involved in the regulation of HIF1α pathway in breast cancer [26]. Furthermore, hypoxia has also been identified as an ER stress inducer in various cancers, and thus, both hypoxia and ERS play an essential role in cancer angiogenesis [42, 43, 48], their interactions in cancers should receive greater attention. In our study, we determined that the expression of PDIA4 is regulated by the transcriptional activity of XBP1 in GBM cells under ER stress, rather than by HIF1α under hypoxic conditions. Nonetheless, XBP1 has been reported to be upregulated by hypoxia in HT1080 cells [49]. Thus, the role of the HIF1α/XBP1 axis in the regulation of GBM progression, and especially in the regulation of angiogenesis, remains an interesting topic.

In the animal studies, we chose the human U87 GBM cell to establish xenograft GBM model using nude mice, the cross reactivities between human derived VEGFA and mouse endothelial cells might raise attentions. Although there are species differences between human and mouse, some previous researches and our study have proved the cross reactivities exactly existing. In a previous study, authors implanted human A673 rhabdomyosarcoma and G55 glioblastoma multiforme cells in the nude mice, and they found anti-VEGFA treatment (human VEGFA) can decrease the density of vascular elements in the xenograft tumor region of mice [50]. Besides, a series publications reported similar researches [51–54], implanting human cancer cell lines into nude mice, and their results also proved the strong cross activities between human VEGFA and mouse endothelial cells.

## Conclusions

**Fig. 7:**
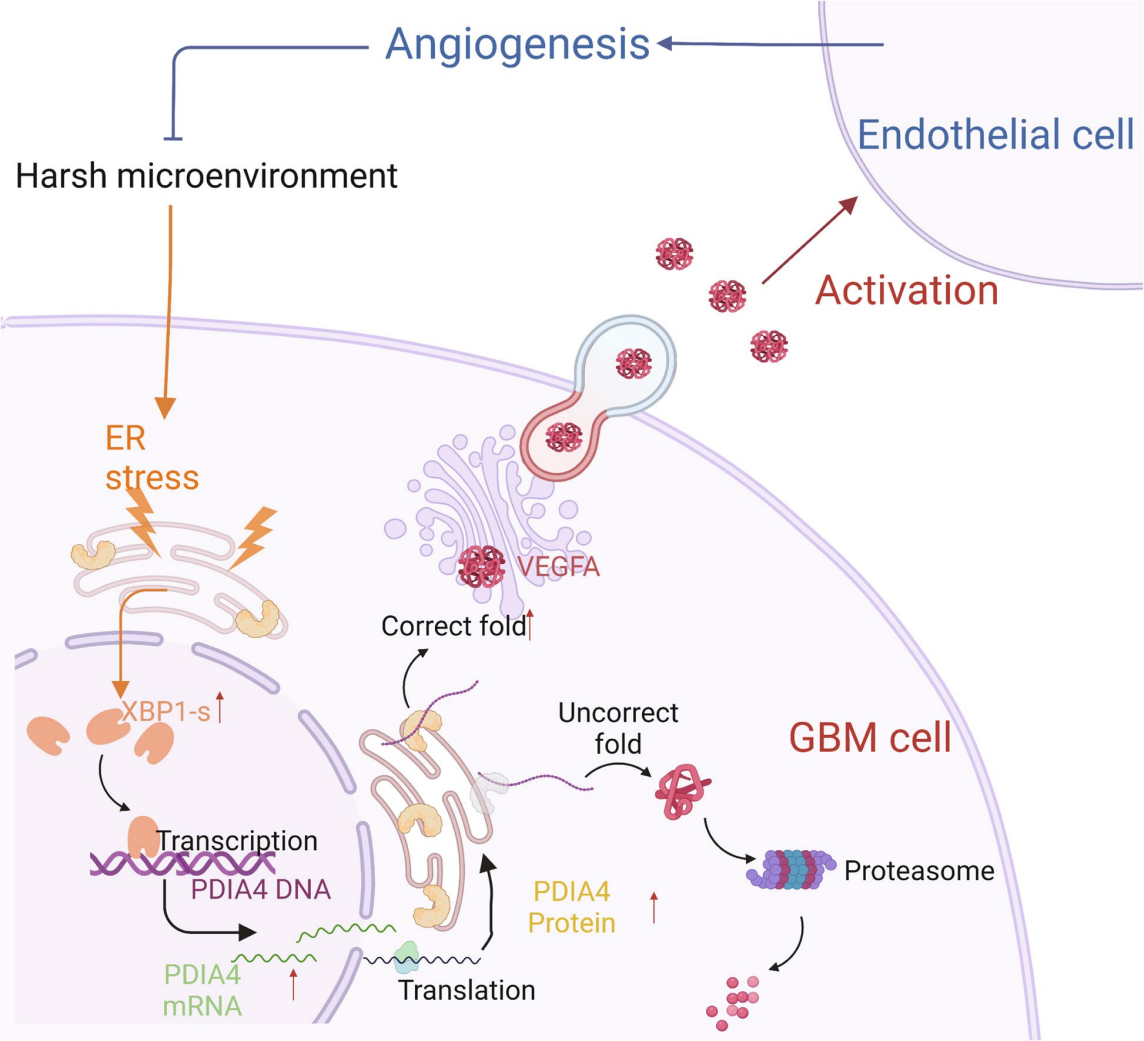
Mechanism diagram of XBP1/PDIA4/VEGFA axis in GBM. Under the extracellular stress, ER stress of GBM cell would start, and activates the unfolded protein pathway (URP), which will upregulate the X-box binding protein 1 (XBP1), and then transcriptionally upregulated PDIA4 expression. As an ER stress effect protein, PDIA4 could increase the expression and secretion of VEGFA protein in GBM cells, and activate the tumor associated endothelial cell. This mechanism elicits the pro-angiogenesis function of PDIA4 of GBM

In this study, we proved PDIA4 is a pro-angiogenesis regulator in GBM. PDIA4 is not only a genomically amplified gene, but it can also be transcriptionally up-regulated by the ER-stress regulator XBP1. It regulates GBM angiogenesis by controlling VEGFA secretion via the oxidoreductase activities of its CXXC motifs. The relative mechanism pattern was concluded and diagramed in the Fig. 7. Furthermore, PDIA4 expression is also associated with the level of prop-edema in GBM patients, and higher expression of PDIA4 xenograft GBM present greater resistance to antiangiogenetic therapy. Our study points to the biological role of PDIA4 in GBM angiogenesis, and its association with resistance to antiangiogenetic therapy. These findings indicate that PDIA4 can serve as an effective biomarker and target for precision antiangiogenetic therapy in patients with GBM.

## Supplementary Information

**Additional file 1: Figure S1.** (A-C) Kaplan-Meier survival analysis of three public GBM datasets (including two cohorts from CGGA datasets, and one cohort from GEO repository) validated the robust prognostic role of PDIA4 in GBM patients. (D) Protein expressions of PDIA4 in human astrocyte and five GBM cell lines. (E) Validation of PDIA4 protein expression levels in lentivirus transfected U87 and LN229 cells.

**Additional file 2: Figure S2.** (A) Metascape enrichment analysis of DEGs between low- and high-PDIA4 GBM subgroups in the TCGA-GBM bulk RNA-seq dataset. (B) Mass spectrogram of FMDVYQR peptide sequence identified in PDIA4 protein substrates. (C) Intracellular VEGFA protein expressions are upregulated in PDIA4 overexpression U87 cells and downregulated in PDIA4-knockdown LN229 cells.

**Additional file 3: Figure S3.** (A) The three potential transcriptional factors of PDIA4 (ATF6, XBP1 and HIF1A) are all significantly upregulated in GBMs compared with normal human tissues. ***P < 0.001. (B) The expressions of all the three potential transcriptional factors (ATF6, XBP1 and HIF1A) are positive correlated with the PDIA4 expression in GBMs. (C) The GSEA analysis indicates the enrichment of hypoxia in hig-PDIA4 GBMs. (D) Public ChIP dataset revealed there is a significant binding site of HIF1α on PDIA4 promoter in T47D cells but not in PC-3 cells. (E) Western blot data unveiled that PDIA4 protein expressions are not upregulated when we cultured the U87 and LN229 cells with 0% O2.

**Additional file 4: Supplementary Table 1.** Secretory protein identified in Tao et al.'s work

**Additional file 5: Supplementary Table 2.** The substrate proteins of PDIA4 we identified

## Data Availability

All data are available in the main text or the supplementary materials.

## Abbreviations

PDIA4: Protein disulfide-isomerase A4
ERS: Endoplasmic reticulum stress
GBM: Glioblastoma
MS: Mass spectrum
ELISA: Enzyme-linked immunosorbent assay
RT-qPCR: Real-time quantitative polymerase chain reaction
VEGFA: Vascular endothelial growth factor A
XBP1: X-box binding protein 1
OS: Overall survival
PFS: Progression-free survival
UPR: Unfolded protein response
TMZ: Temozolomide
TM: Tunicamycin
ChIP: Chromatin immunoprecipitation
BEV: Bevacizumab

## References

[1] Killock D (2015). CNS cancer: molecular classification of glioma. Nat Rev Clin Oncol.

[2] Jiang T, Nam DH, Ram Z, Poon WS, Wang J, Boldbaatar D (2021). Clinical practice guidelines for the management of adult diffuse gliomas. Cancer Lett.

[3] Brennan CW, Verhaak RG, McKenna A, Campos B, Noushmehr H, Salama SR (2013). The somatic genomic landscape of glioblastoma. Cell.

[4] Aldape K, Zadeh G, Mansouri S, Reifenberger G, von Deimling A (2015). Glioblastoma: pathology, molecular mechanisms and markers. Acta Neuropathol.

[5] Xu S, Sankar S, Neamati N (2014). Protein disulfide isomerase: a promising target for cancer therapy. Drug Discov Today.

[6] Tufo G, Jones AW, Wang Z, Hamelin J, Tajeddine N, Esposti DD (2014). The protein disulfide isomerases PDIA4 and PDIA6 mediate resistance to cisplatin-induced cell death in lung adenocarcinoma. Cell Death Differ.

[7] Lee E, Lee DH (2017). Emerging roles of protein disulfide isomerase in cancer. BMB Rep.

[8] Kim TW, Ryu HH, Li SY, Li CH, Lim SH, Jang WY (2017). PDIA6 regulation of ADAM17 shedding activity and EGFR-mediated migration and invasion of glioblastoma cells. J Neurosurg.

[9] Wang M, Zhang W, Liu Y, Ma Z, Xiang W, Wen Y (2022). PDIA4 promotes glioblastoma progression via the PI3K/AKT/m-TOR pathway. Biochem Biophys Res Commun.

[10] Ye L, Park JJ, Dong MB, Yang Q, Chow RD, Peng L (2019). In vivo CRISPR screening in CD8 T cells with AAV-Sleeping Beauty hybrid vectors identifies membrane targets for improving immunotherapy for glioblastoma. Nat Biotechnol.

[11] Chiavari M, Ciotti GMP, Canonico F, Altieri F, Lacal PM, Graziani G (2020). PDIA3 Expression in Glioblastoma Modulates Macrophage/Microglia Pro-Tumor Activation. Int J Mol Sci.

[12] Wang Z, Zhang H, Cheng Q (2020). PDIA4: The basic characteristics, functions and its potential connection with cancer. Biomed Pharmacother.

[13] Meunier L, Usherwood YK, Chung KT, Hendershot LM (2002). A subset of chaperones and folding enzymes form multiprotein complexes in endoplasmic reticulum to bind nascent proteins. Mol Biol Cell.

[14] Xing F, Song Z, Cheng Z (2022). High expression of PDIA4 promotes malignant cell behavior and predicts reduced survival in cervical cancer. Oncol Rep.

[15] Yin F, Yi S, Wei L, Zhao B, Li J, Cai X (2019). Microarray-based identification of genes associated with prognosis and drug resistance in ovarian cancer. J Cell Biochem.

[16] Chen TY, Yang CY, Yang MT, Kuo TF, Chang CL, Chen CL (2022). Protein disulfide isomerase a4 promotes lung cancer development via the Stat3 pathway in stromal cells. Clin Transl Med.

[17] Kuo TF, Chen TY, Jiang ST, Chen KW, Chiang YM, Hsu YJ (2017). Protein disulfide isomerase a4 acts as a novel regulator of cancer growth through the procaspase pathway. Oncogene.

[18] Qian S, Zhang S, Wu Y, Ding Y, Shen, Li X (2020). Protein Disulfide Isomerase 4 Drives Docetaxel Resistance in Prostate Cancer. Chemotherapy.

[19] N , Cancer Genome Atlas Research (2008). Comprehensive genomic characterization defines human glioblastoma genes and core pathways. Nature.

[20] Zhao Z, Zhang KN, Wang Q, Li G, Zeng F, Zhang Y (2021). Chinese Glioma Genome Atlas (CGGA): A Comprehensive Resource with Functional Genomic Data from Chinese Glioma Patients. Genomics Proteomics Bioinformatics.

[21] Gravendeel LA, Kouwenhoven MC, Gevaert O, de Rooi JJ, Stubbs AP, Duijm JE (2009). Intrinsic gene expression profiles of gliomas are a better predictor of survival than histology. Cancer Res.

[22] Darmanis S, Sloan SA, Croote D, Mignardi M, Chernikova S, Samghababi P (2017). Single-Cell RNA-Seq Analysis of Infiltrating Neoplastic Cells at the Migrating Front of Human Glioblastoma. Cell Rep.

[23] Schneider CA, Rasband WS, Eliceiri KW (2012). NIH Image to ImageJ: 25 years of image analysis. Nat Methods.

[24] Ritchie ME, Phipson B, Wu D, Hu Y, Law CW, Shi W (2015). limma powers differential expression analyses for RNA-sequencing and microarray studies. Nucleic Acids Res.

[25] Zhou Y, Zhou B, Pache L, Chang M, Khodabakhshi AH, Tanaseichuk O (2019). Metascape provides a biologist-oriented resource for the analysis of systems-level datasets. Nat Commun.

[26] Liang H, Xiao J, Zhou Z, Wu J, Ge F, Li Z (2018). Hypoxia induces miR-153 through the IRE1alpha-XBP1 pathway to fine tune the HIF1alpha/VEGFA axis in breast cancer angiogenesis. Oncogene.

[27] Varghese F, Bukhari AB, Malhotra R, De A (2014). IHC Profiler: an open source plugin for the quantitative evaluation and automated scoring of immunohistochemistry images of human tissue samples. PLoS ONE.

[28] Carpentier G, Berndt S, Ferratge S, Rasband W, Cuendet M, Uzan G (2020). Angiogenesis Analyzer for ImageJ - A comparative morphometric analysis of "Endothelial Tube Formation Assay" and "Fibrin Bead Assay". Sci Rep.

[29] Liu T, Ortiz JA, Taing L, Meyer CA, Lee B, Zhang Y (2011). Cistrome: an integrative platform for transcriptional regulation studies. Genome Biol.

[30] Castro-Mondragon JA, Riudavets-Puig R, Rauluseviciute I, Lemma RB, Turchi L, Blanc-Mathieu R (2022). JASPAR 2022: the 9th release of the open-access database of transcription factor binding profiles. Nucleic Acids Res.

[31] Singh VK, Mangalam AK, Dwivedi S, Naik S (1998). Primer premier: program for design of degenerate primers from a protein sequence. Biotechniques.

[32] Oka OB, Pringle MA, Schopp IM, Braakman I, Bulleid NJ (2013). ERdj5 is the ER reductase that catalyzes the removal of non-native disulfides and correct folding of the LDL receptor. Mol Cell.

[33] Mathieu V, De Neve N, Le Mercier M, Dewelle J, Gaussin JF, Dehoux M (2008). Combining bevacizumab with temozolomide increases the antitumor efficacy of temozolomide in a human glioblastoma orthotopic xenograft model. Neoplasia.

[34] Jessop CE, Chakravarthi S, Garbi N, Hammerling GJ, Lovell S, Bulleid NJ (2007). ERp57 is essential for efficient folding of glycoproteins sharing common structural domains. EMBO J.

[35] Camargo LL, Babelova A, Mieth A, Weigert A, Mooz J, Rajalingam K (2013). Endo-PDI is required for TNFalpha-induced angiogenesis. Free Radic Biol Med.

[36] Wilkinson B, Gilbert HF (2004). Protein disulfide isomerase. Biochim Biophys Acta.

[37] Liu T, Jia P, Ma H, Reed SA, Luo X, Larman HB (2017). Construction and Screening of a Lentiviral Secretome Library. Cell Chem Biol.

[38] Shoulders MD, Ryno LM, Genereux JC, Moresco JJ, Tu PG, Wu C (2013). Stress-independent activation of XBP1s and/or ATF6 reveals three functionally diverse ER proteostasis environments. Cell Rep.

[39] Wu J, Chen S, Liu H, Zhang Z, Ni Z, Chen J (2018). Tunicamycin specifically aggravates ER stress and overcomes chemoresistance in multidrug-resistant gastric cancer cells by inhibiting N-glycosylation. J Exp Clin Cancer Res.

[40] Zhang X, Yuan Y, Jiang L, Zhang J, Gao J, Shen Z (2014). Endoplasmic reticulum stress induced by tunicamycin and thapsigargin protects against transient ischemic brain injury: Involvement of PARK2-dependent mitophagy. Autophagy.

[41] Jain R, Poisson LM, Gutman D, Scarpace L, Hwang SN, Holder CA (2014). Outcome prediction in patients with glioblastoma by using imaging, clinical, and genomic biomarkers: focus on the nonenhancing component of the tumor. Radiology.

[42] Chen X, Cubillos-Ruiz JR (2021). Endoplasmic reticulum stress signals in the tumour and its microenvironment. Nat Rev Cancer.

[43] Wang M, Kaufman RJ (2014). The impact of the endoplasmic reticulum protein-folding environment on cancer development. Nat Rev Cancer.

[44] Wick W, Gorlia T, Bendszus M, Taphoorn M, Sahm F, Harting I (2017). Lomustine and Bevacizumab in Progressive Glioblastoma. N Engl J Med.

[45] Kreisl TN, Kim L, Moore K, Duic P, Royce C, Stroud I (2009). Phase II trial of single-agent bevacizumab followed by bevacizumab plus irinotecan at tumor progression in recurrent glioblastoma. J Clin Oncol.

[46] Friedman HS, Prados MD, Wen PY, Mikkelsen T, Schiff D, Abrey LE (2009). Bevacizumab alone and in combination with irinotecan in recurrent glioblastoma. J Clin Oncol.

[47] Baysan M, Woolard K, Cam MC, Zhang W, Song H, Kotliarova S (2017). Detailed longitudinal sampling of glioma stem cells in situ reveals Chr7 gain and Chr10 loss as repeated events in primary tumor formation and recurrence. Int J Cancer.

[48] Kaur B, Khwaja FW, Severson EA, Matheny SL, Brat DJ, Van Meir EG (2005). Hypoxia and the hypoxia-inducible-factor pathway in glioma growth and angiogenesis. Neuro Oncol.

[49] Romero-Ramirez L, Cao H, Nelson D, Hammond E, Lee AH, Yoshida H (2004). XBP1 is essential for survival under hypoxic conditions and is required for tumor growth. Cancer Res.

[50] Kim KJ, Li B, Winer J, Armanini M, Gillett N, Phillips HS (1993). Inhibition of vascular endothelial growth factor-induced angiogenesis suppresses tumour growth in vivo. Nature.

[51] Feng H, Jin Z, Liang J, Zhao Q, Zhan L, Yang Z (2021). FOXK2 transcriptionally activating VEGFA induces apatinib resistance in anaplastic thyroid cancer through VEGFA/VEGFR1 pathway. Oncogene.

[52] Feng H, Liu K, Shen X, Liang J, Wang C, Qiu W (2020). Targeting tumor cell-derived CCL2 as a strategy to overcome Bevacizumab resistance in ETV5(+) colorectal cancer. Cell Death Dis.

[53] Luo Y, Yang Z, Yu Y, Zhang P (2022). HIF1alpha lactylation enhances KIAA1199 transcription to promote angiogenesis and vasculogenic mimicry in prostate cancer. Int J Biol Macromol.

[54] Han H, Lin T, Wang Z, Song J, Fang Z, Zhang J (2023). RNA-binding motif 4 promotes angiogenesis in HCC by selectively activating VEGF-A expression. Pharmacol Res.

