# Supplementary material for: Protein disulfide-isomerase A4 confers glioblastoma angiogenesis promotion capacity and resistance to anti-angiogenic therapy
Source: J Exp Clin Cancer Res. 2023 Mar 30;42:77. doi: 10.1186/s13046-023-02640-1 (PMC10061982; doi:10.1186/s13046-023-02640-1)
Supplement: Supplementary file 1 — Additional file 1: Figure S1. (A-C) Kaplan-Meier survival analysis of three public GBM datasets (including two cohorts from CGGA datasets, and one cohort from GEO repository) validated the robust prognostic role of PDIA4 in GBM patients. (D) Protein expressions of PDIA4 in human astrocyte and five GBM cell lines. (E) Validation of PDIA4 protein expression levels in lentivirus transfected U87 and LN229 cells. [file 13046_2023_2640_MOESM1_ESM.pdf]

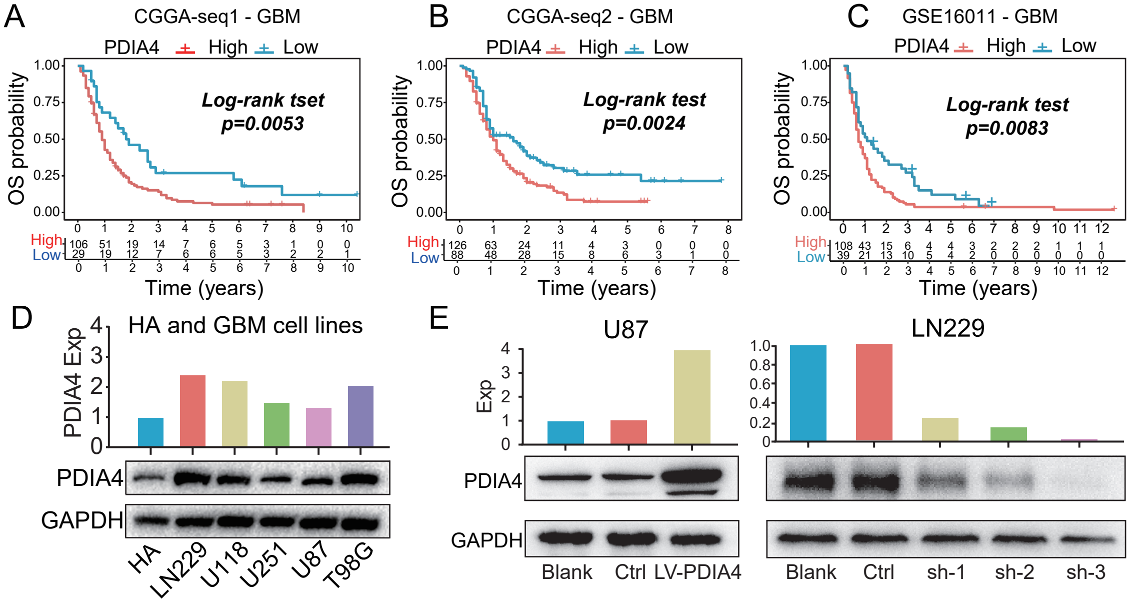

A
CGGA-seq1 - GBM
PDIA4 High Low
Log-rank tset
p=0.0053
OS probability
Time (years)
B
CGGA-seq2 - GBM
PDIA4 High Low
Log-rank test
p=0.0024
C
GSE16011 - GBM
PDIA4 High Low
Log-rank test
p=0.0083
D
HA and GBM cell lines
PDIA4 Exp
PDIA4
GAPDH
HA
LN229
U118
U251
U87
T98G
E
U87
LN229
Exp
Blank
Ctrl
LV-PDIA4
sh-1
sh-2
sh-3
