# Supplementary figures and images for: Protein disulfide-isomerase A4 confers glioblastoma angiogenesis promotion capacity and resistance to anti-angiogenic therapy
Source: J Exp Clin Cancer Res. 2023 Mar 30;42:77. doi: 10.1186/s13046-023-02640-1 (PMC10061982; doi:10.1186/s13046-023-02640-1)

A

## TCGA-GBM

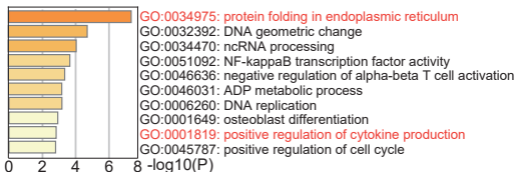

B

## MS peaks of FMDVYQR peptide

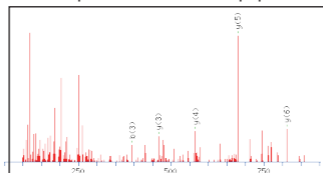

C

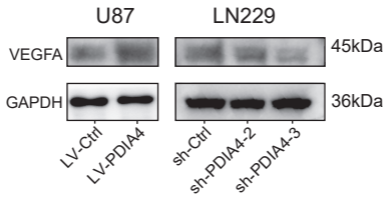

Supplement: Supplementary file 2 — Additional file 2: Figure S2. (A) Metascape enrichment analysis of DEGs between low- and high-PDIA4 GBM subgroups in the TCGA-GBM bulk RNA-seq dataset. (B) Mass spectrogram of FMDVYQR peptide sequence identified in PDIA4 protein substrates. (C) Intracellular VEGFA protein expressions are upregulated in PDIA4 overexpression U87 cells and downregulated in PDIA4-knockdown LN229 cells. [file 13046_2023_2640_MOESM2_ESM.pdf]

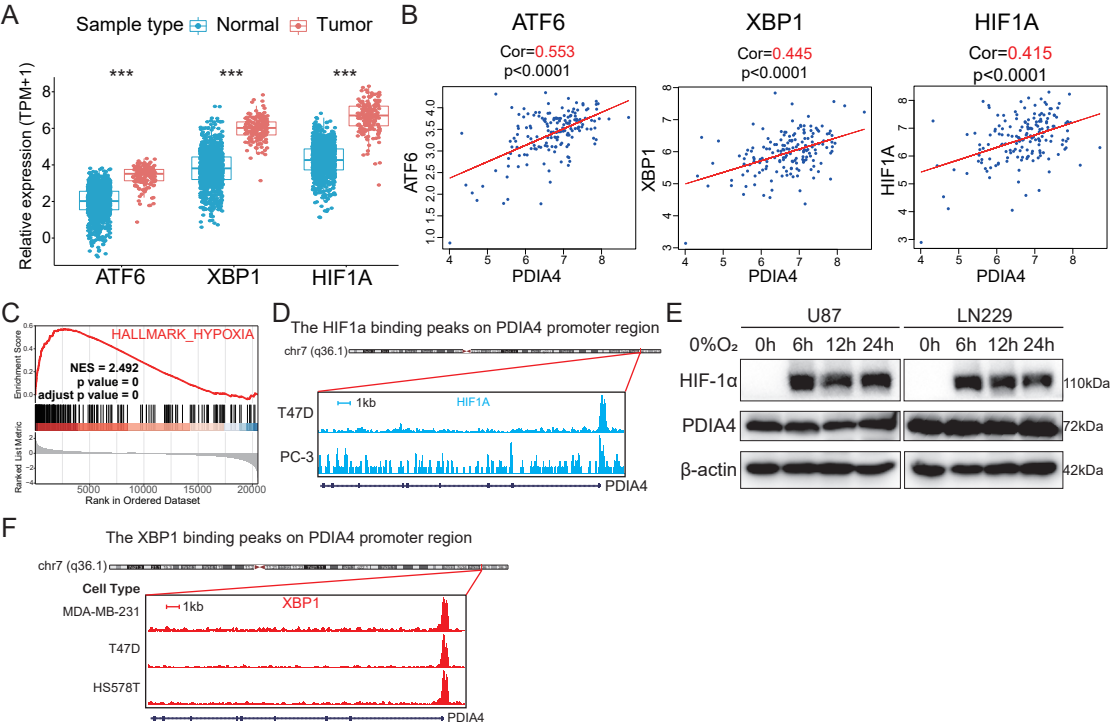

Supplement: Supplementary file 3 — Additional file 3: Figure S3. (A) The three potential transcriptional factors of PDIA4 (ATF6, XBP1 and HIF1A) are all significantly upregulated in GBMs compared with normal human tissues. ***P < 0.001. (B) The expressions of all the three potential transcriptional factors (ATF6, XBP1 and HIF1A) are positive correlated with the PDIA4 expression in GBMs. (C) The GSEA analysis indicates the enrichment of hypoxia in hig-PDIA4 GBMs. (D) Public ChIP dataset revealed there is a significant binding site of HIF1α on PDIA4 promoter in T47D cells but not in PC-3 cells. (E) Western blot data unveiled that PDIA4 protein expressions are not upregulated when we cultured the U87 and LN229 cells with 0% O2. [file 13046_2023_2640_MOESM3_ESM.pdf]
